# Alterations in Antioxidant Micronutrient Concentrations in Placental Tissue, Maternal Blood and Urine and the Fetal Circulation in Pre-eclampsia

**DOI:** 10.3390/ijms24043579

**Published:** 2023-02-10

**Authors:** Lesia O. Kurlak, Paula J. Scaife, Louise V. Briggs, Fiona Broughton Pipkin, David S. Gardner, Hiten D. Mistry

**Affiliations:** 1 School of Medicine (Stroke Research), University of Nottingham, Nottingham NG7 2UH, UK; 2 Clinical, Metabolic and Molecular Physiology Research Group, University of Nottingham, Derby DE22 3DT, UK; 3 School of Engineering, University of Nottingham, Nottingham NG7 2RD, UK; 4 Department of Obstetrics & Gynaecology, University of Nottingham, Nottingham NG5 1PB, UK; 5 School of Veterinary Medicine and Science, University of Nottingham, Loughborough LE12 5RD, UK; 6 Department of Women and Children’s Health, School of Life Course and Population Sciences, King’s College London, London SE1 1UL, UK

**Keywords:** hypertension in pregnancy, nutrition, antioxidant micronutrients, placenta

## Abstract

Trace elements such as selenium and zinc are vital components of many enzymes, including endogenous antioxidants, and can interact with each other. Women with pre-eclampsia, the hypertensive disease of pregnancy, have been reported as having changes in some individual antioxidant trace elements during pregnancy, which are related to maternal and fetal mortality and morbidity. We hypothesised that examination of the three compartments of (a) maternal plasma and urine, (b) placental tissue and (c) fetal plasma in normotensive and hypertensive pregnant women would allow identification of biologically significant changes and interactions in selenium, zinc, manganese and copper. Furthermore, these would be related to changes in the angiogenic markers, placental growth factor (PlGF) and Soluble Fms-Like Tyrosine Kinase-1 (sFlt-1) concentrations. Venous plasma and urine were collected from healthy non-pregnant women (n = 30), normotensive pregnant controls (n = 60) and women with pre-eclampsia (n = 50) in the third trimester. Where possible, matched placental tissue samples and umbilical venous (fetal) plasma were also collected. Antioxidant micronutrient concentrations were measured by inductively coupled plasma mass-spectrometry. Urinary levels were normalised to creatinine concentration. Plasma active PlGF and sFlt-1 concentrations were measured by ELISA. Maternal plasma selenium, zinc and manganese were all lower in women with pre-eclampsia (*p* < 0.05), as were fetal plasma selenium and manganese (*p* < 0.05 for all); maternal urinary concentrations were lower for selenium and zinc (*p* < 0.05). Conversely, maternal and fetal plasma and urinary copper concentrations were higher in women with pre-eclampsia (*p* < 0.05). Differences in placental concentrations varied, with lower overall levels of selenium and zinc (*p* < 0.05) in women with pre-eclampsia. Maternal and fetal PlGF were lower and sFlt-1 higher in women with pre-eclampsia; maternal plasma zinc was positively correlated with maternal plasma sFlt-1 (*p* < 0.05). Because of perceptions that early- and late-onset pre-eclampsia have differing aetiologies, we subdivided maternal and fetal data accordingly. No major differences were observed, but fetal sample sizes were small following early-onset. Disruption in these antioxidant micronutrients may be responsible for some of the manifestations of pre-eclampsia, including contributing to an antiangiogenic state. The potential benefits of mineral supplementation, in women with deficient intakes, during pregnancy to reduce pre-eclampsia remain an important area for experimental and clinical research.

## 1. Introduction

Complicating between 2 and 10% of pregnancies worldwide, pre-eclampsia is a pregnancy specific syndrome characterised by new-onset hypertension and maternal, fetal and/or placental complications in the second half of pregnancy [1]. It is associated with the greatest risk of maternal and fetal morbidity and mortality of all pregnancy-related hypertensive disorders [2]. For example, the risk of stillbirth at 34 weeks’ gestation is 7-fold higher in women with pre-eclampsia than in normotensive women and the risk increases exponentially the earlier the pre-eclampsia occurs [3]. Moreover, hypertensive disorders of pregnancy are also associated with an increased risk of long-term cardiovascular and metabolic morbidity and mortality for mother and child [4,5].

Pregnancy places substantial demands on a woman’s stores of trace elements, including those implicated in antioxidant function [6,7]. Differences in both absolute and proportional concentrations of various trace elements have been reported between normotensive and hypertensive pregnant women and their babies [7]. Pregnancy imposes a three-compartment structure on the metabolism of these elements (maternal, placental and fetal), with the maternal compartment being markedly influenced by her residual renal function. Furthermore, placental transfer of micronutrients can be passive, facilitated, active or by pinocytosis. Thus, simple spot measurement of maternal plasma concentrations of any substance can be broadly uninformative in the context of disturbed placental function, such as occurs in pre-eclampsia, and not be reflective of concentrations in the fetal compartment.

Vast changes occur within women’s physiology during pregnancy to support the additional requirements of her growing fetus. Deficiencies/alterations of specific antioxidant activities associated with the micronutrients selenium, copper, zinc and manganese can result in poor pregnancy outcomes, including fetal growth restriction and pre-eclampsia [6,8,9,10,11].

Selenium is an essential cofactor for the antioxidant enzyme, glutathione peroxidase (GPx), crucial for scavenging damaging free radicals and thus modulating cellular oxidant stress and redox-mediated responses. We and others have previously demonstrated lower serum selenium concentrations and GPx enzyme activity, associated with elevated oxidative stress in pre-eclampsia [8,12,13]. Zinc is required for the function of over 300 enzymes and 1000 transcription factors and promotes antioxidant activity. Several reports have suggested that zinc deficiency may be associated with increased incidence of pre-eclampsia [6,7,14,15]. Manganese is also an important cofactor for a number of enzymes, including the antioxidant manganese superoxide dismutase (Mn-SOD), which may protect the placenta from oxidative stress, by detoxifying superoxide anions [16]. Circulating manganese concentrations are lower in women giving birth to babies with fetal growth restriction, indicating that this micronutrient may be important in maintaining fetal growth [17]. Manganese is one of the least studied of the micronutrients; a small retrospective study of African American mothers reported reduced umbilical cord whole blood manganese concentrations in neonates born to mothers with pre-eclampsia [18]. A recent prospective pregnant cohort study reported higher first trimester manganese red blood cell concentration, with a lower risk of pre-eclampsia, with the effect increasing in a dose–response manner [19].

Copper is an essential cofactor for a number of enzymes involved in metabolic reactions, angiogenesis, oxygen transport and antioxidant protection, including catalase, superoxide dismutase (SOD) and cytochrome oxidase [20]. It is, however, a double-edged sword; retrospective studies from Turkey have shown elevation of maternal serum copper levels in pre-eclampsia after clinical onset of the disease [11,21]. In addition, our data from the screening for pregnancy endpoints (SCOPE) study found raised maternal copper concentrations in women at 15 weeks’ gestation, who subsequently went on to develop pre-eclampsia [22].

Supplementation trials with antioxidant micronutrients have been conflicting [6]. A previous study (not adequately powered due to a statistical error), supplementing women with 60 µg/d (as selenium-enriched yeast), or placebo treatment between 12 and 14 weeks of gestation until delivery, reported a reduction in the antiangiogenic marker, soluble fms-like tyrosine kinase-1 (sFlt-1) [23]. Further detailed studies regarding these micronutrients are required prior to any further supplementation trials.

Although several studies have examined maternal circulating micronutrients in relation to pre-eclampsia, this is the first study that has measured them and the angiogenic markers placental growth factor (PlGF) and sFlt-1, in all three compartments (e.g., paired maternal and fetal plasma, maternal urine and placental tissue levels). We hypothesised that deficiencies in selenium, zinc and manganese, as well as increased copper concentrations in plasma and placental samples, will be found in women with pre-eclampsia. In addition, altered levels in urine, reflecting abnormal renal function, as is often found in women with pre-eclampsia, will also be observed. We suggest such a pattern of micronutrients will contribute to the known oxidative stress that women with pre-eclampsia experience.

## 2. Results

### 2.1. Participants

Baseline demographic and pregnancy outcome data are presented in Table 1. All women in this study were white European. As can be seen, by definition, women who had pre-eclampsia had significantly higher blood pressures (*p* < 0.05), with the pre-eclamptic women also having significant proteinuria. Overall, the groups were matched for maternal age, BMI and parity.

### 2.2. Selenium

**Figure 1:**
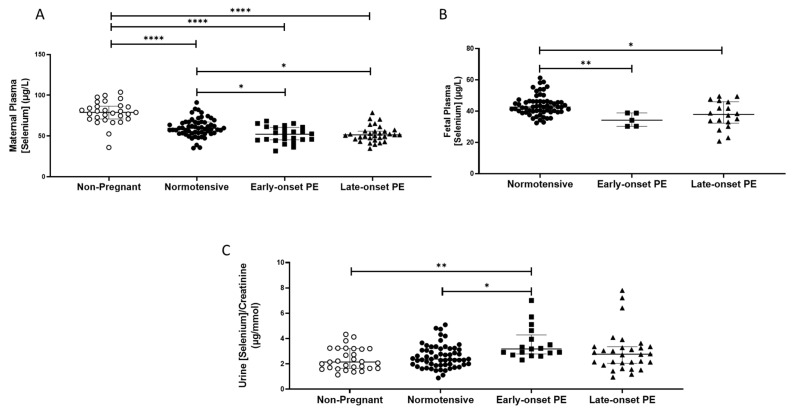
(**A**) Maternal plasma selenium concentrations in non-pregnant (n = 30) and normotensive pregnant women (n = 60) and those with early- (n = 24) and late-onset (n = 31) pre-eclampsia (PE); (**B**) umbilical cord venous (fetal) plasma selenium concentrations in normotensive pregnant women (n = 60) and those with early- (n = 5) and late-onset (n = 18) PE; (**C**) Urinary selenium/creatinine concentrations in non-pregnant (n = 28) and normotensive pregnant women (n = 60) and women with early- (n = 23) and late-onset (n = 31) PE. Data presented as median (IQR); * *p* < 0.05; ** *p* < 0.005; **** *p* < 0.0001.

The non-pregnant women had higher plasma selenium concentrations (median (IQR); µg/L: 78.9 (70.9, 86.7)) compared to all pregnant groups (*p* < 0.0001 for all; Figure 1A). Maternal plasma concentrations were significantly lower in women who had pre-eclampsia (51.4 (45.1, 60.2)), compared to normotensive controls (59.5 (53.7, 66.9); *p* < 0.0001). When the pre-eclampsia group was split by early-/late-onset-pre-eclampsia, the differences remained (*p* = 0.001), with lower selenium concentrations in both early- (*p* = 0.03) and late- (*p* = 0.01) onset pre-eclampsia, compared to normotensive controls (Figure 1A).

Urinary selenium/creatinine concentrations did not differ between non-pregnant women (2.2 (1.7, 3.2) µg/mmol) and normotensive pregnant women (2.4 (1.9, 3.2); *p* > 0.05), but lower concentrations were observed compared to women who had pre-eclampsia (2.9 (2.1, 3.4); *p* < 0.05). On splitting the pre-eclampsia group, only the early-onset pre-eclampsia had higher selenium/creatinine concentrations compared to normotensive non-pregnant controls (*p* < 0.05; Figure 1C).

Feetal plasma selenium concentrations at delivery were lower than in maternal samples (Figure 1B) and were further reduced in babies of women who had pre-eclampsia (37.4 (31.7, 41.8)), compared to normotensive controls (42.7 (39.5, 46.2); *p* < 0.0001). When the pre-eclampsia group was further split into early-/late-onset pre-eclampsia, the differences remained (*p* = 0.001); lower selenium concentrations were noted in the plasma of babies born to women that had early- or late-onset pre-eclampsia (*p* < 0.05 for both), compared to normotensive controls (Figure 1B).

When analysing placental selenium concentrations (Table 2), samples collected from the periphery were lower in the pre-eclampsia group compared to normotensive control placentae (*p* = 0.032). When further split, both the periphery and near cord placentae were different between groups (*p* = 0.027 and *p* = 0.012 respectively; Table 2), with late-onset pre-eclampsia samples being lower than normotensive controls (*p* = 0.007 for both; Table 2). Moreover, for the near cord samples, there was also a significant difference between the early- and late-onset pre-eclampsia, with the latter group being lower (*p* = 0.026; Table 2).

### 2.3. Zinc

**Figure 2:**
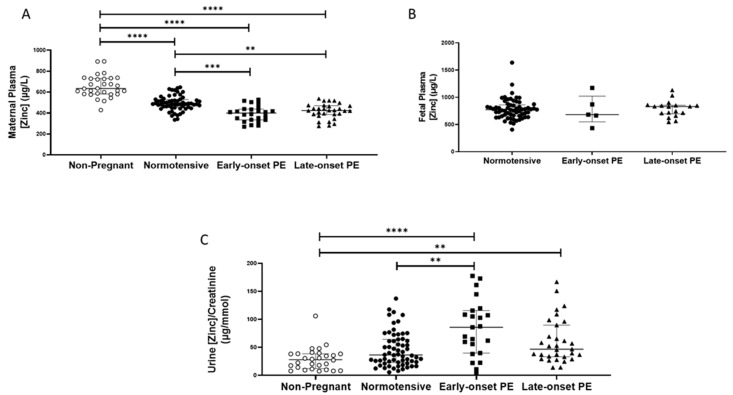
(**A**) Maternal plasma zinc concentrations in non-pregnant (n = 30) and normotensive pregnant women (n = 60) and those with early- (n = 24) and late-onset (n = 31) pre-eclampsia (PE); (**B**) umbilical cord venous (fetal) plasma zinc concentrations in normotensive pregnant women (n = 60) and those with early- (n = 5) and late-onset (n = 18) PE; (**C**) Urinary zinc/creatinine concentrations in non-pregnant (n = 28), normotensive pregnant (n = 60) and women with early- (n = 23) and late-onset (n = 31) PE. Data presented as median (IQR); ** *p* < 0.005; *** *p* < 0.001; **** *p* < 0.0001.

As with selenium, non-pregnant women (median (IQR); µg/L: 634.8 (581.8, 730.1)), had higher plasma zinc concentrations compared to all other pregnant groups (*p* < 0.0001 for all; Figure 2A). Nevertheless, maternal plasma concentrations were significantly lower in women who had pre-eclampsia (418.3 (368.5, 453.9)), compared to normotensive controls (486.3 (454.6, 526.7); *p* < 0.0001). When the pre-eclampsia group was split by early-/late-onset pre-eclampsia, the differences remained (*p* < 0.0001), with lower zinc concentrations in both early- (*p* = 0.0002) and late-onset (*p* = 0.004) pre-eclampsia, compared to normotensive controls (Figure 2A); median concentrations were, however, similar between the two pre-eclamptic groups.

Median urinary zinc/creatinine concentrations were similar in non-pregnant women and normotensive pregnant controls but were considerably lower than in women who had pre-eclampsia ((27.9 (12.0, 38.4), compared with non-pregnant women 57.5 (36.8, 103.9) µg/mmol *p* < 0.0001). Moreover, higher levels were found in women who had pre-eclampsia, compared to normotensive pregnant women (36.3 (23.2, 64.0); *p* = 0.003). On splitting the pre-eclampsia group, only women with early-onset pre-eclampsia had higher zinc/creatinine concentrations compared to normotensive pregnant women (*p* = 0.009; Figure 2C).

Fetal plasma zinc concentrations (Figure 2B), were considerably higher than maternal concentrations in all groups, but levels were not different between babies of women who had pre-eclampsia (830.2 (672.9, 855.5)), compared to normotensive controls (771.7 (660.1, 856.2); *p* > 0.05), nor were there concentration differences when the pre-eclampsia group was split by early-/late-onset-pre-eclampsia, (*p* > 0.05; Figure 2B).

Placental zinc concentrations in samples from the periphery were lower in the women who had pre-eclampsia compared with normotensive controls (*p* = 0.02). No differences were seen for either the middle or near cord samples (*p* = 0.08 and *p* = 0.34, respectively). When sub-grouped, again, only the periphery placentae appeared lower in both the early- and late-onset pre-eclampsia groups, but they did not quite reach significance (*p* = 0.05; Table 2).

### 2.4. Manganese

**Figure 3:**
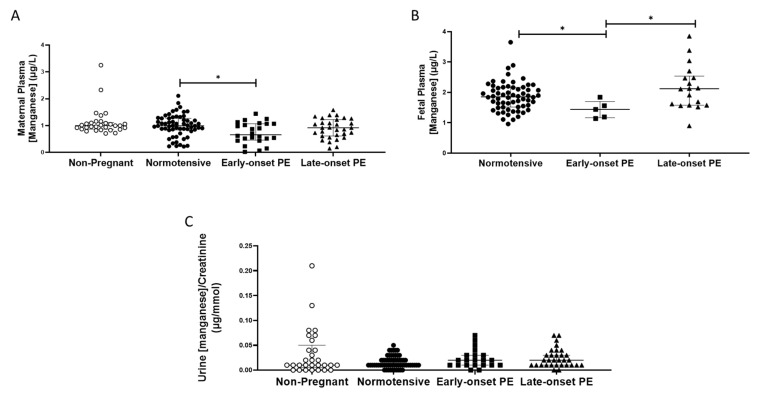
(**A**) Maternal plasma manganese concentrations in non-pregnant (n = 30), normotensive pregnant women (n = 60) and those with early- (n = 24) and late-onset (n = 31) pre-eclampsia (PE); (**B**) umbilical cord venous (fetal) plasma manganese concentrations in normotensive pregnant women (n = 60) and those with early- (n = 5) and late-onset (n = 18) PE; (**C**) Urinary manganese/creatinine concentrations in non-pregnant (n = 28), normotensive pregnant women (n = 60) and those with early- (n = 23) and late-onset (n = 31) PE. Data presented as median (IQR); * *p* < 0.05.

Plasma manganese concentrations were unchanged in pregnancy by comparison with those non-pregnant women, except that woman with early-onset pre-eclampsia had somewhat lower concentrations than those in normotensive pregnant women (0.85 (0.55, 1.1), compared with normotensive controls (1.0 (0.84,1.27) µg/L; *p* < 0.05; Figure 3A).

No differences were found between any of the groups for urinary manganese/creatinine concentrations (*p* > 0.05; Figure 3C). Women with pre-eclampsia had higher urinary manganese output (0.2 (0.01, 0.3)) compared to normotensive controls (0.1 (0.1, 0.2); *p* = 0.04). However, when split by pre-eclampsia group, no differences were seen between groups (*p* > 0.05; Figure 3C).

Fetal plasma manganese concentrations were higher than maternal samples; no differences were seen between babies born to normotensive controls (1.85 (1.52, 5.16)) and those born after pre-eclampsia (1.84 (1.56, 2.38); *p* > 0.05). However, when sub-grouped, the early-onset pre-eclampsia samples had lower manganese concentrations compared to both the normotensive controls (*p* = 0.03) and late-onset pre-eclampsia samples (*p* = 0.02; Figure 3B).

Placental manganese concentrations did not differ either between groups, even after sub-grouping between early- and late-onset pre-eclampsia, for all 3 locations (*p* > 0.05 for all; Table 2).

### 2.5. Copper

**Figure 4:**
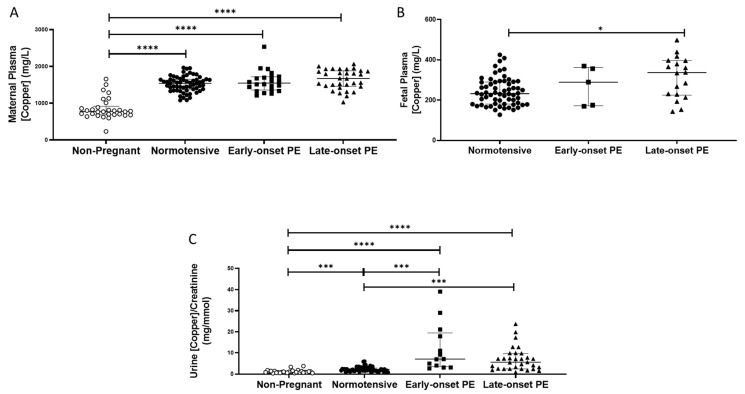
(**A**) Maternal plasma copper concentrations in non-pregnant (n = 30), normotensive pregnant women (n = 60) and those with early- (n = 24) and late-onset (n = 31) pre-eclampsia (PE); (**B**) umbilical cord venous (fetal) plasma copper concentrations in normotensive pregnant women (n = 60) and those with early- (n = 5) and late-onset (n = 18) PE; (**C**) Urinary copper/creatinine concentrations in non-pregnant (n = 28), normotensive pregnant women (n = 60) and those with early- (n = 23) and late-onset (n = 31) PE. Maternal plasma copper levels were significantly higher overall in women with pre-eclampsia than in normotensive women, but only increased in late-onset PE, compared to normotensive controls when spilt. Data presented as median (IQR); * *p* < 0.05; *** *p* < 0.001; **** *p* < 0.0001.

Non-pregnant women had lower plasma copper concentrations (median (IQR); mg/L: 778 (685, 918)), compared to all pregnant groups (*p* < 0.0001 for all; Figure 4A). Maternal plasma concentrations were significantly higher overall in women who had pre-eclampsia (1606 (1451, 1856)), compared to normotensive controls (1541 (1383, 1645); *p* = 0.04), but did not differ between early and late-onset pre-eclampsia.

Non-pregnant women had significantly lower urinary copper/creatinine levels (0.93 (0.66, 1.40 mg/mmol)) compared to all pregnant groups (*p* < 0.001; Figure 4C). Three-fold higher concentrations were found overall in women who had pre-eclampsia (6.8 (2.8, 9.8)), compared to normotensive pregnant controls (2.2 (1.6, 2.8); *p* < 0.0001; Figure 4C). On splitting the pre-eclampsia group, both those with early- and late-onset pre-eclampsia had higher copper/creatinine concentrations compared to normotensive controls (*p* < 0.001 for both; Figure 4C).

Fetal plasma copper concentrations were markedly lower than maternal plasma. They were higher in samples from babies of pre-eclamptic women (334.9 (221.1, 374.4)), compared to normotensive controls (232.4 (180.8, 289.5); *p* = 0.02). When the pre-eclampsia group was split by early-/late-onset-pre-eclampsia, the differences remained (*p* = 0.044), with higher copper concentrations only between late-onset pre-eclampsia (*p* = 0.013), compared to normotensive controls (Figure 4B).

Placental copper concentrations did not differ either between groups or by sampling location (*p* > 0.05 for all; Table 2).

### 2.6. PlGF and sFlt-1

**Figure 5:**
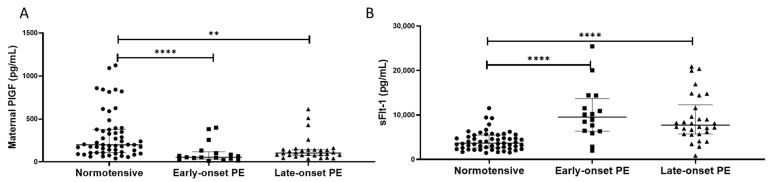
(**A**) Maternal plasma placental growth factor (PlGF) concentrations and (**B**) maternal plasma soluble fms-like tyrosine kinase-1 (sFlt-1) concentrations in normotensive pregnant women (n = 55) and those with early- (n = 17) and late-onset (n = 30) pre-eclampsia (PE). Data presented as median (IQR); ** *p* < 0.005; **** *p* < 0.0001.

Maternal plasma PlGF concentrations were lower in women with pre-eclampsia (median (IQR) pg/mL: 92.5 (54.0, 143)), compared to normotensive controls (201 (109, 377); *p* < 0.0001). When sub-grouped, lower PlGF concentrations were found in both the early- (55 (39, 102)) and late-onset samples (109 (80, 143)), compared to normotensive controls (*p* < 0.0001 for both; Figure 5A).

In contrast, plasma sFlt-1 concentrations were higher in women with pre-eclampsia (8300 (6194, 13155) pg/mL), compared to normotensive controls (3844 (2591, 5483); *p* < 0.0001). When sub-grouped, higher sFlt-1 concentrations were found in both the early- (10090 (6459, 13115)) and late-onset samples (8111 (5724, 11571)), compared to normotensive controls (*p* < 0.0001 for both; Figure 5B).

### 2.7. Correlations

**Figure 6:**
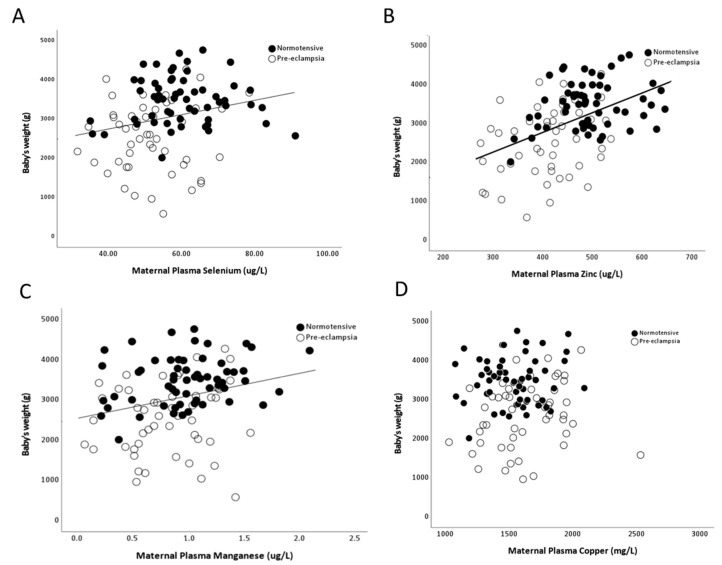
Scatter plots of birthweights in relation to maternal plasma (**A**) selenium (r = 0.29; *p* = 0.002); (**B**) zinc (Zn; r = 0.47; *p* < 0.0001); (**C**) manganese (r = 0.26; *p* = 0.03) and (**D**) copper (r = 0.03; *p* > 0.05).

As there were minimal/no differences by onset of pre-eclampsia, data were pooled for further analysis. Maternal selenium (r = 0.29; *p* = 0.002), zinc (r = 0.47; *p* < 0.0001) and manganese (r = 0.26; *p* = 0.03), but not copper (r = 0.03; *p* > 0.05), concentrations positively correlated with birthweights (Figure 6A–D).

**Figure 7:**
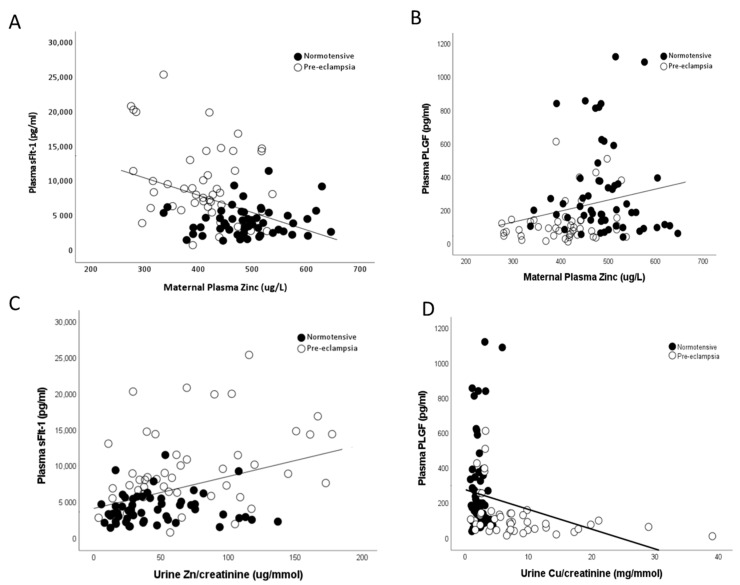
Scatter plots of maternal plasma Zn with (**A**) maternal plasma soluble fms-like tyrosine kinase-1 (sFlt-1; r = −0.359; *p* < 0.0001) and (**B**) maternal plasma placental growth factor (PlGF; r = 0.257; *p* = 0.01). Urine Cu/creatinine with (**C**) maternal plasma sFlt-1 (r = 0.559; *p* < 0.0001) and (**D**) maternal plasma PlGF (r = −0.467; *p* < 0.0001).

Maternal plasma zinc concentrations were also found to be negatively correlated to measured maternal sFlt-1 (r = −0.359; *p* < 0.0001; Figure 7A) and positively associated with PlGF (r = 0.257; *p* = 0.01; Figure 7B) levels. Conversely, urine copper/creatinine levels were positively correlated to maternal sFlt-1 (r = 0.559; *p* < 0.0001; Figure 7C) and negatively correlated with PlGF (r = −0.467; *p* < 0.0001; Figure 7D) levels.

**Figure 8:**
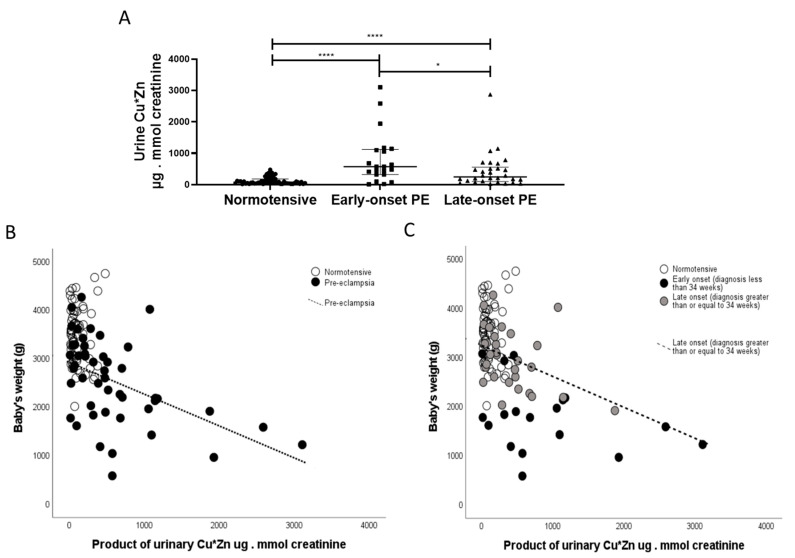
The product of CuxZn; µg/L in urine were determined in samples from (**A**) normotensive pregnant (n = 60) and women with early- (n = 24) and late-onset (n = 31) pre-eclampsia (PE). Data are presented corrected for urine flow by determination of urine creatinine as median (IQR); * *p* < 0.05; **** *p* < 0.0001. Scatter plots of CuxZn with (**B**) with birthweight in pre-eclampsia samples (r = −4.70; *p* < 0.0001; dotted line) and (**C**) when spilt by early-/late-onset pre-eclampsia, the negative trends were seen only in the late-onset pre-eclampsia group (r = −4.50; *p* = 0.01; hashed line).

Finally, in exploratory analyses, based on our data from non-pregnant patients with acute kidney injury (AKI) [24], a positive correlation was observed between urinary copper and urinary zinc (r = 0.450; *p* < 0.0001). Furthermore, the product of urinary CuxZn was also significantly greater in women with pre-eclampsia versus normotensive controls (*p* < 0.0001). When sub-grouped by early-/late-onset pre-eclampsia, both were higher than normotensive controls (*p* < 0.0001 for both; Figure 8A); with the early-onset having higher products than the late-onset pre-eclampsia group (*p* = 0.031; Figure 8A). A negative association was found between this with birthweight only in the pre-eclampsia group (r = −5.20; *p* < 0.0001; Figure 8B). When spilt by early-/late-onset pre-eclampsia, the negative trends were seen only in the pre-eclampsia groups and only significant in the late-onset pre-eclampsia group (r = −4.50; *p* = 0.01; Figure 8C).

## 3. Discussion

This study illustrates key alterations in concentrations of trace elements essential for antioxidant function, in paired (where possible) maternal and fetal plasma, plus maternal urine and placental tissue in women diagnosed with pre-eclampsia. As it is becoming increasingly evident that there are aetiologic differences between those women who had early-/late-onset pre-eclampsia, we included sub-group analysis of the women who had pre-eclampsia. The reduced maternal and fetal plasma selenium observed is in accordance with previous data from our group and others [8,12,25]. Moreover, increased urinary selenium concentrations in women with pre-eclampsia suggest the renal handling of selenium is also adversely affected. Although we are not aware of any detailed studies on renal handling of selenium in human pregnancy, one published study of rats with nephrotoxic serum nephritis, an induced model of acute kidney injury, similarly reported lower kidney selenium concentration [26]. In this study, plasma selenium concentrations, even in the non-pregnant women, were lower than recommended levels [27], as we have also previously shown in the UK population [8], presumably reflecting inadequate dietary intake. Reduced maternal selenium levels could unfavourably affect the functional activities of the antioxidant GPx, compromising systemic and cellular protection against oxidative stress [12]. Babies generally have lower plasma selenium concentrations than mothers, implying that selenium is transported via the placenta down a concentration gradient. Decreased placental selenium concentrations in women with pre-eclampsia could reflect underlying placental damage. This may reduce availability to both the placenta and the circulation, leading to the inadequate antioxidant levels and subsequent oxidative stress characteristic of pre-eclampsia [28], not only in the mothers but also, potentially, in the fetus. The lowest levels were observed in samples from women with late-onset pre-eclampsia. Late-onset pre-eclampsia is thought to stem more from underlying maternal factors than early-onset disease. We suggest that there is a greater synthesis of the antioxidant selenonprotein, GPx in such women throughout pregnancy, thus reducing selenium stores [8,29]. The differences in placental selenium concentrations between standardised placental sampling sites were as predicted. We previously reported graded differences in GPx3 (higher in the inner region) and GPx4 (higher in the periphery), as well as placental GPx enzyme activity being reduced in tissue from pre-eclamptic women as compared to normotensive women, the difference being more pronounced nearest cord insertion [28].

Zinc deficiency has increased over the last decade due to a trend towards zinc-poor diets, based on processed foods and soy-based substitutes, as well as food grown in zinc-poor soil [30]. Zinc-deficient rats and mice have been shown to have evidence of increased oxidative damage [31]. Alteration in zinc homeostasis may have adverse effects on pregnancy outcome, including prolonged labour, fetal growth restriction or embryonic or fetal death [6,32]. Zinc deficiency has been associated with pre-eclampsia since the 1980s [9,10,12], with the lower serum zinc concentrations in mothers who develop pre-eclampsia being suggested to be at least partly due to reduced oestrogen and zinc binding-protein levels [33]. Similar to this study, others have reported lower maternal zinc concentrations in women with pre-eclampsia, suggesting that the reduction may not only affect antioxidant protection, but could also contribute to the rise in blood pressure via increased lipid peroxidation [14] and cytoplasmic zinc acting in sensory nerves, endothelium and smooth muscle [34]. Urine zinc excretion is a reliable biomarker for zinc status [35]; hence, lower urine zinc concentrations as observed in our women with pre-eclampsia may therefore reflect lower zinc intake in these women. Unlike other micronutrients, such as iron, there is no active storage of zinc in the body, to be drawn upon when intakes are inadequate [36]. A highly effective homeostatic mechanism responds to alterations in zinc intake, upregulating absorption and conserving losses via the gastrointestinal tract and kidneys when intakes fall [35]. Any potential renal impairment associated with pre-eclampsia, particularly women with early-onset pre-eclampsia, may therefore disrupt this precise homeostasis.

Zinc is transported across the placenta via active transport, via transporters such as zinc importer protein (ZIP) and zinc exporter protein (ZNT), families from the mother to the fetus [37]. Similarly to our data, a previous study reported higher zinc concentrations in the fetal compartment relative to the maternal, even when a diagnosis of pre-eclampsia might reduce maternal levels [10]. The lack of difference in placental zinc concentrations may be due to the fetus ensuring adequate levels are maintained, through placental transport or retention of zinc through an unspecified mechanism. A previous study on pregnant women found that the PlGF/sFlt-1 ratio positively corrected with zinc intake in the first trimester [38]. This further supports our observed associations of maternal zinc concentrations with both PlGF and sFLt-1, suggesting that zinc may play a contributing role in dysregulation of the angiogenic balance in pre-eclampsia.

Manganese is one of the least studied micronutrients at present, and we are not aware of any supplementation trials that have been published, which may reflect the lack of data on manganese concentrations in pregnancy. However, a study of 1274 women from the Boston Birth Cohort measured red blood cell manganese between 24 and 72 h after delivery and found that after multivariable adjustment, a 1 SD increment in manganese was associated with 32% lower risk of developing pre-eclampsia (PR = 0.68; 95% CI, 0.54–0.86) [39]. This concurs with the lower levels of maternal plasma manganese found in women with pre-eclampsia in the current study. In addition, higher fetal plasma manganese concentrations concur with the suggestion that placental transport of manganese probably involves a combination of active and passive transport processes [40,41]. Furthermore, the lower maternal and fetal levels in those with early-onset pre-eclampsia, suggest that manganese may also indirectly contribute to the dysregulation of the maternal immune system, as has previously been reported [42]. The higher urine manganese in women with pre-eclampsia reflects alterations in renal handling and inadequate re-absorption of this element, but it is not known whether this is a primary or secondary effect.

Copper concentration is known to be higher in maternal relative to fetal (e.g., umbilical cord) plasma [43,44,45], and is confirmed by our data (Figure 4). It has been suggested that the placenta acts as a blockade for the transfer of copper from the mother to the fetus [45,46]; copper is only transported across the placenta by diffusion [45,47]. It is thought that as copper is a redox-active transition metal and can participate in single electron reactions and catalyse the formation of free radicals, including undesirable hydroxyl radicals, higher cellular levels could contribute to increased oxidative stress, characteristic of pre-eclampsia [11]. Therefore, while copper is an integral component of an antioxidant system (Cu/Zn SOD), even modest increases in free cellular copper may lead to the production of reactive oxygen species and oxidative stress, which has been linked to the development of pre-eclampsia [48]. However, we have suggested women developing pre-eclampsia may increase the uptake of essential metals like copper as seen in this current study, in an attempt to buffer the higher levels of oxidative stress associated with the disease and thus, may be the result of pre-eclampsia [22]. The higher urine copper levels could also relate to the angiogenic imbalance characteristic of pre-eclampsia; urinary copper has been shown to be associated with higher circulating antiangiogenic sFlt-1 levels [48]. This concurs with the current study where higher urine copper was associated with higher maternal plasma sFlt-1 and inversely associated with PlGF concentrations. It is also of interest that the product of urinary CuxZn, being significantly higher in women with pre-eclampsia, is similar to an experimental study of AKI in a porcine model and in intensive care patients with a diagnosis of AKI post-ICU admission [24,49]. It is hypothesised that pre-eclampsia is a pre-renal cause of AKI and elevation of these urinary trace elements in women with pre-eclampsia adds further weight to this argument. The negative correlation between birthweight with urinary CuxZn, further confirms the association of both copper and zinc with fetal growth and requires further investigation to establish potential mechanisms. Mineral biomarkers in urine have many advantages for possible earlier diagnosis of renal dysfunction, as they are stable at room temperature for long periods of time and are less likely to be affected by various comorbidities such as proteinuria or glucosuria. Further work is needed to determine whether urinary trace elements may predict development of PE in later pregnancy.

Positive associations between maternal plasma selenium, zinc and manganese with birthweight suggests that these micronutrients may be contributing factors for adequate fetal growth, whether directly or through other properties such as antioxidant effects. Selenium deficiency has previously been linked with several reproductive complications, including small-for-gestational-age infants [12,50,51,52,53]. Furthermore, zinc supplementation during pregnancy has been reported to significantly increase birthweight and head circumference [54], highlighting the importance of adequate zinc supply during pregnancy. A recent metabolomics study of maternal plasma at 36 weeks of gestation found increased copper concentrations in women who delivered small-for-gestational-age infants [55]. Moreover, negative associations between urinary CuxZn with birthweight suggests that any possible maternal renal dysfunction (as bio-marked through increased appearance of these trace elements in urine), may have further consequences on fetal growth.

A limitation of this study was that detailed dietary intakes and sociodemographic status data were not collected to further confirm intakes and potential confounders. Future large studies are needed to provide information on this. However, all women in this study were white European, so in broad terms were comparable. In addition, we appreciate that the number of fetal samples we had were small, and thus, future work is required to confirm the findings from the current study.

In conclusion, the data presented here suggest that maternal micronutrient status is increasingly recognised to have an important role in the health and wellbeing of pregnant women and their developing baby. Alteration to the levels of these antioxidant micronutrients may be responsible for some of the manifestations of pre-eclampsia, including contributing to an antiangiogenic state. The potential benefits of supplementation, or strategies focusing on providing nutritional guidance, specifically to pregnant women, will be pivotal in helping to ensure optimal health of both mother and baby and thus, remain an important area for experimental and clinical research.

## 4. Materials and Methods

### 4.1. Cohort and Sample Collection

Fully informed, signed consent for participation was obtained from all women, following HRA-REC ethics committee approval of the study gained by the University of Nottingham (REF: 15/EM/0523). All procedures involving the participants were in accordance with the Helsinki Declaration of 1975 and samples were collected between 2016 and 2021. Pre-eclampsia (n = 55) was defined as systolic blood pressure ≥140 mmHg and diastolic blood pressure ≥90 mmHg, determined on 2 occasions >4 h apart and arising after 20 weeks of gestation in a previously normotensive woman. This hypertension was accompanied with *de novo* proteinuria (protein: creatinine ratio (PCR) > 30; urine protein concentration > 3 g/L in 2 random clean-catch midstream specimens, collected >4 h apart), with no evidence of urinary tract infection [56]. No women had any underlying renal or hypertensive disease before 20 weeks’ gestation or fetal growth restriction. For sub-group analysis, the pre-eclampsia group was further split by early- (diagnosis ≤34 weeks; n = 24) and late- (diagnosis > 34 weeks; n = 31)) onset pre-eclampsia [57].

Healthy normotensive pregnant women (n = 60) matched for age were also recruited, who had no complications and no evidence of any urinary tract infections. Finally, healthy non-pregnant women (n = 30), also age-matched with the pregnant women, were recruited. All non-pregnant women were using the combined oral contraceptive pill. Medical and obstetric histories, including delivery data, were obtained from each woman. A summary of the demographic and pregnancy outcomes of the women recruited in this study are presented in Table 1.

Maternal venous blood samples and urine were collected before delivery; where possible, umbilical venous (fetal) blood samples were collected immediately after delivery. Samples were processed as previously described [8,58] and stored as 250 µL aliquots at −80 °C until analysis. Full depth placental tissue samples (spanning the maternal and fetal sides and removing any decidual tissue), were collected, when possible, from three standardised locations between cord insertion and placental border (1 cm from the cord insertion (near cord), 1 cm from the periphery (periphery), and midway between the two (middle)), avoiding placental infarcts. The placental samples were taken within 10 min of delivery, membranes removed, and tissue washed in ice cold 1× PBS to remove maternal blood contamination. Samples were snap frozen in liquid nitrogen and stored at −80 °C.

### 4.2. Measurement of Micronutrient Content in Maternal Plasma, Urine and Placenta

Plasma and urine concentrations of copper, zinc, selenium and manganese were assayed by Inductively Coupled Plasma Mass Spectrometry (ICP-MS) as previously described in detail [53]. Quality of analysis was assured by the use of the reference materials Seronorm and UTAK (Nycomed Pharma AS, Zürich, Switzerland); limits of detection have been published previously [59]. Trace-element-free techniques were used during collection and analysis, following guidelines from the International Zinc Nutrition Consultative Group. Both intra- and inter-assay coefficients of variation were <5%.

Placental tissue concentrations of the same micronutrients (mmol/g of dry matter (DM)) were also measured by ICP-MS (intra-assay variability < 2%), after prior digestion of ~400 mg of freeze-dried tissue (further allowing determination of percentage water content) and subsequent digestion using 2% nitric acid as previously described [49,59,60]. Certified reference material (NIST SRM bovine liver, 1577c) was used to validate elemental recovery and to correct for any batch variation.

### 4.3. Measurement of PlGF and sFlt-1

Maternal plasma samples were analysed using ELISAs according to manufacturer’s instructions. PlGF (#DPG00, Bio-Techne, detection limit of 7 pg/mL) and sFlt-1 (#SVR100B, Bio-Techne, detection limit of 3.5 pg/mL), in undiluted samples.

### 4.4. Statistical Analysis

All tests were performed using SPSS version 27 and GraphPad Prism version 9. Summary data are presented as means ± standard deviation (SD) or median and interquartile range (IQR) as appropriate. The Kruskal–Wallis test, followed by Mann–Whitney U-test, was used for multiple group analysis. The Student’s *t*-test or Mann–Whitney U-tests were applied depending on whether the data distribution was normal or skewed, as indicated by the Kolmogorov–Smirnov test. Spearman’s Rank correlation test was used to test associations. The null hypothesis was rejected where *p* < 0.05.

## Figures and Tables

**Table 1:** Demographic, clinical and biochemical data of participants.

Parameter	Non-Pregnant (n = 30)	Normotensive (n = 60)	Early-Onset Pre-eclampsia (n = 24)	Late-Onset Pre-eclampsia (n = 31)
Maternal age (years)	27 ± 7.6	32 ± 5.4	33 ± 5.4	29 ± 6.4
Booking body mass index (kg/m^2^)	24.7 ± 4.7	27.3 ± 6.3	32.3 ± 8.0	29.1 ± 6.8
Nulliparous (n (%))	17 (57)	31 (52)	13 (54)	17 (55)
Max. systolic blood pressure outside labour at booking (mmHg)	124 ± 13.1	128 ± 12.7	163 ± 10.0 **	154 ± 9.0 **
Max. diastolic blood pressure outside labour at booking (mmHg)	78 ± 9.3	80 ± 8.1	103 ± 6.8 **	99 ± 5.8 **
Protein:creatinine ratio (g/mmol)	-	-	177 (80, 378)	127 (58, 269)
Gestation age at delivery (weeks)	-	39.1 ± 1.1	32.7 ± 2.8 *	38.4 ± 1.8 *
Birthweight (kg)	-	3.50 (2.99, 3.88)	1.77 (1.29, 2.04) *	2.99 (2.49, 3.48) *
Caesarean Sections (n (%)	-	46 (76)	16 (66)	17 (50)

* *p* < 0.05; ** *p* < 0.0001 between both the non-pregnant and normotensive with the pre-eclampsia diagnostic groups. Data presented as mean ± SD or median (IQR).

**Table 2:** Placental micronutrient concentrations *.

Location of Biopsy	Normotensive Control (n = 60)	Early-Onset Pre-eclampsia (n = 5)	Late-Onset Pre-eclampsia (n = 13)
**Selenium (** **µ** **g/Kg)**			
Periphery	**1.03 [0.98, 1.09] ^a^**	**1.05 [1.01, 1.08]**	**0.95 [0.87, 1.02] ^a^**
Middle	1.02 [0.94, 1.07]	0.98 [0.91, 1.03]	0.91 [0.88, 0.93]
Near Cord	**1.01 [0.95, 1.05] ^a^**	**1.02 [1.01, 1.20] ^b^**	**0.91 [0.88, 0.93] ^a,b^**
**Zinc (** **µ** **g/Kg)**			
Periphery	58.6 [52.7, 62.4]	52.2 [52.1, 58.2]	55.8 [52.6, 62.4]
Middle	56.9 [52.1, 62.1]	54.6 [44.0, 55.5]	55.3 [53.7, 58.7]
Near Cord	56.6 [52.5, 61.8]	51.2 [45.9, 55.1]	56.7 [54.2, 61.2]
**Manganese (** **µ** **g/Kg)**			
Periphery	0.48 [0.37, 0.56]	0.48 [0.41, 0.58]	0.47 [0.41, 0.58]
Middle	0.44 [0.34, 0.52]	0.35 [0.33, 0.39]	0.39 [0.33, 0.43]
Near Cord	0.46 [0.36, 0.57]	0.37 [0.31, 0.41]	0.46 [0.31, 0.63]
**Copper (mg/Kg)**			
Periphery	5.48 [4.79, 6.17]	5.19 [4.73, 6.06]	5.02 [4.93, 5.86]
Middle	5.03 [4.55, 5.53]	4.70 [4.04, 5.24]	5.33 [4.86, 6.58]
Near Cord	5.06 [4.59, 5.57]	5.05 [4.59, 5.79]	5.16 [4.57, 5.48]

^a^*p* < 0.05 between normotensive and late-onset pre-eclampsia; ^b^ *p* < 0.05 between early-onset and late-onset pre-eclampsia; key significances have been highlighted in bold. Placental zinc levels were significantly higher overall in women with pre-eclampsia than in normotensive women. However, the differences between early or late-onset pre-eclampsia did not reach statistical significance. * Data presented as median [IQR].

## Data Availability

Data are contained within the article and additional raw data can be obtained from the corresponding author on request.
